# Defining the genetic basis of early onset hereditary spastic paraplegia using whole genome sequencing

**DOI:** 10.1007/s10048-016-0495-z

**Published:** 2016-09-28

**Authors:** Kishore R Kumar, G.M. Wali, Mahesh Kamate, Gautam Wali, André E Minoche, Clare Puttick, Mark Pinese, Velimir Gayevskiy, Marcel E Dinger, Tony Roscioli, Carolyn M. Sue, Mark J Cowley

**Affiliations:** 1 Department of Neurogenetics, Kolling Institute of Medical Research, Royal North Shore Hospital and University of Sydney, St Leonards, 2065 Australia; 2 Kinghorn Centre for Clinical Genomics, Garvan Institute of Medical Research, Darlinghurst, Australia; 3 Neurospecialities Centre, Belgaum, India; 4 Department of Paediatrics, KLE University’s Jawaharlal Nehru J N Medical College, Belgaum, India; 5 St Vincent’s Clinical School, University of New South Wales, Sydney, Australia; 6 Department of Medical Genetics, Sydney Children’s Hospital, Randwick, Australia

**Keywords:** Hereditary spastic paraplegia, Whole genome sequencing, Metabolic, Gangliosidosis, Zellweger, SPG54, SPG56

## Abstract

We performed whole genome sequencing (WGS) in nine families from India with early-onset hereditary spastic paraplegia (HSP). We obtained a genetic diagnosis in 4/9 (44 %) families within known HSP genes (*DDHD2* and *CYP2U1*), as well as perixosomal biogenesis disorders (*PEX16*) and GM1 gangliosidosis (*GLB1*). In the remaining patients, no candidate structural variants, copy number variants or predicted splice variants affecting an extended candidate gene list were identified. Our findings demonstrate the efficacy of using WGS for diagnosing early-onset HSP, particularly in consanguineous families (4/6 diagnosed), highlighting that two of the diagnoses would not have been made using a targeted approach.

**Electronic supplementary material:**

The online version of this article (doi:10.1007/s10048-016-0495-z) contains supplementary material, which is available to authorized users.

## Introduction

The hereditary spastic paraplegias (HSPs) are a group of disorders characterised by progressive lower limb weakness and spasticity. There is marked genetic heterogeneity with over 60 genes identified. Massively parallel sequencing approaches such as targeted multigene panels, whole exome sequencing (WES), and whole genome sequencing (WGS) have facilitated genetic diagnosis in HSP [1, 2]. WGS may have advantages over WES including more consistent coverage and the potential for more precise detection of structural variants (SVs) and copy number variants (CNVs). For example, single- or multiexon-sized deletions can be found in autosomal dominant and autosomal recessive HSP (SPG4 and SPG11, respectively). The goal of this study was to assess the utility of WGS in a previously undiagnosed sample of early-onset HSP from India.

## Brief methods

The research was approved by the appropriate institutional ethics committee (HREC/10/HAWKE/132), and all participants provided written informed consent. We initially recruited ten consecutive families with early-onset HSP from the NeuroSpecialities Centre or KLE University Hospital in Belgaum, India.

WGS was performed on at least one affected proband. Parents and siblings were included for consanguineous families, subject to DNA availability. Genomic DNA was extracted from peripheral blood leucocytes (NucleoSpin® Blood, Macherey-Nagel). WGS was performed on the Illumina HiSeq X sequencers at the Kinghorn Centre for Clinical Genomics (KCCG). Data were analysed following the GATK best practises pipeline, as described [3]. Gender and relatedness checks were performed using PLINK and KING [4].

Variants were prioritised according to population frequency databases (including ExAC), variant impact, in silico prediction (SIFT and Polyphen2), known HSP genes (Supplementary Table 1), genes previously associated with neurological phenotypes and the HSPome [5], using *Seave* (seave.bio). For the family studies, variants were also filtered according to homozygous, compound heterozygous, de novo dominant and X-linked recessive models of inheritance, as appropriate. All candidate variants were confirmed by Sanger sequencing. Homozygosity mapping was performed using ROHmer (Puttick et al., manuscript in preparation). Detection of SVs and CNVs was performed using VarPipeSV (Minoche et al., manuscript in preparation). To predict the impact that all coding and intronic SNPs from HSP genes would have on splicing, we used SPANR [6]. Variants were classified according to ACMG 2015 criteria [7]. See the Supplementary File for methods in detail.

## Results

**Fig. 1:**
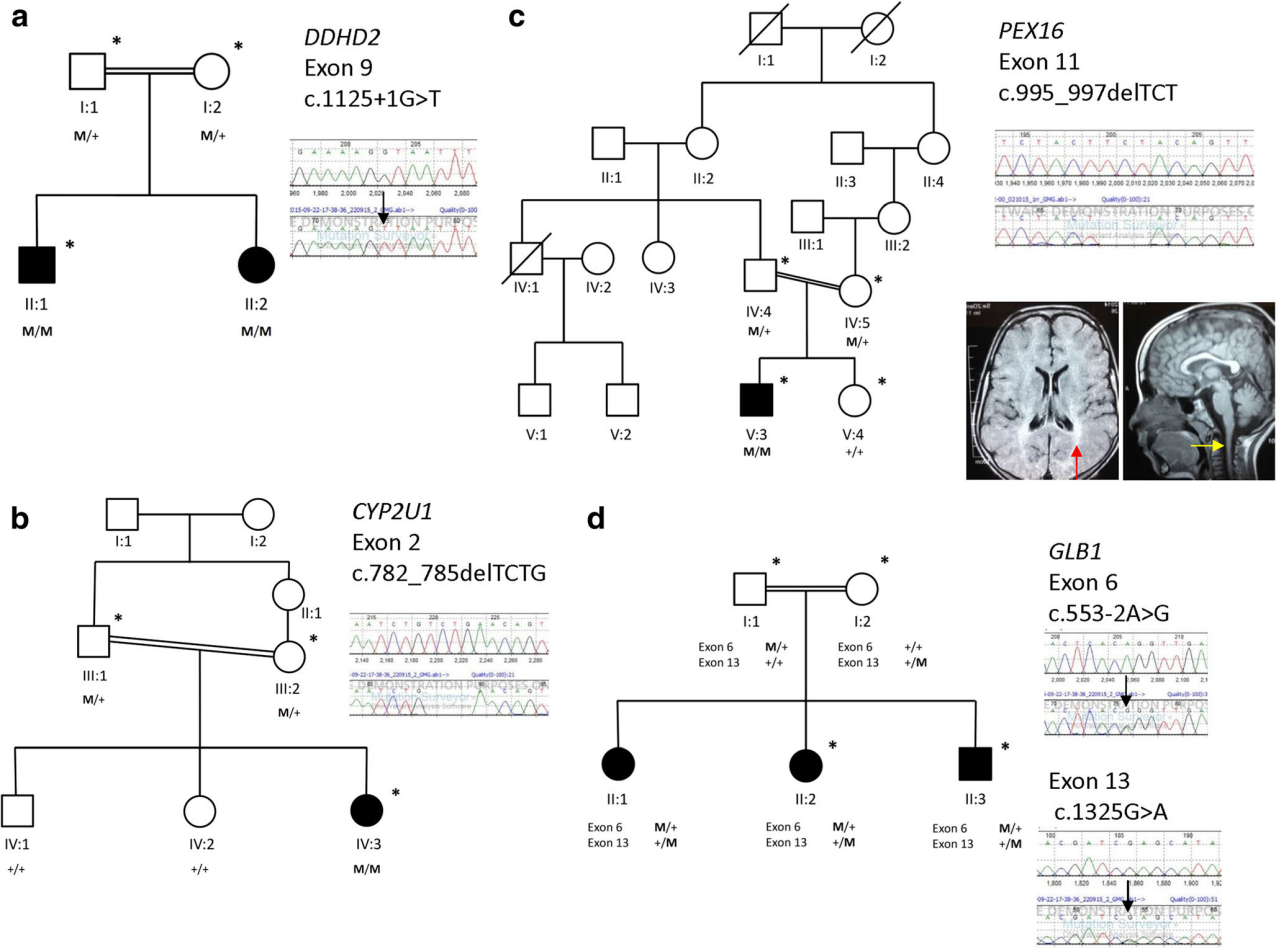
Pedigrees of families with identified putative causal mutations. **a** Family 12 with a homozygous splice site variant in *DDHD2*. **b** Family 9 with a homozygous 4 b.p. deletion in *CYP2U1*. **c** Family 1 with an in-frame deletion in *PEX16*. Inset (*left*) shows transverse MRI brain axial FLAIR sequence showing posterior white matter changes consistent with hypomyelination (*red arrow*). Inset (*right*) demonstrates atrophy of the cervical cord on sagittal T1-weighted MRI brain. **d** Family 7 with compound heterozygous variants in the *GLB1* gene. Electropherograms show wild-type sequence above, sequence in affected children below, *black arrows* indicate missense variants. *Squares*, males; *circles*, females; *diagonal line through symbol*, deceased; *filled symbol*, affected individual; *asterisk*, patient has undergone whole genome sequencing

We recruited ten families to this study; however, following gender and relatedness checks, we identified a sample mixup in family 10, which we could not reconcile; so this family was removed for further consideration (Supplementary Fig. 1). The majority of families studied were consanguineous (6/9) with complex clinical phenotypes (7/9). For the 77 known nuclear HSP genes, 60 (78 %) had 100 % coverage >15× depth, and 69 (90 %) had 95 % coverage >15× depth (Supplementary Fig. 2), giving high power to detect variants. On average, we identified 4.7 M variants in each individual, 6200 of which are in known HSP genes, 49 of which have medium or high impact (Supplementary Table 2). We made a genetic diagnosis of HSP in 4/9 families (Fig. 1, Supplementary Table 3); 2/9 families had mutations in known HSP genes (Supplementary Table 4). Putative disease-causing variants were classified PVS1 or PS1 (ACMG 2015 criteria).

In family 12 (Fig. 1a), we identified a novel homozygous canonical splice site variant in the *DDHD2* gene (NM_015214.2:c.(1125+1G>T)), confirmed to be heterozygous in both unaffected parents. *DDHD2* mutations cause SPG54, which is associated with very early-onset spastic paraplegia (before 2 years of age), intellectual disability, a thin corpus callosum (TCC) and optic nerve involvement [8]. This is consistent with the phenotype in family 12 with infantile-onset spastic paraplegia, cognitive and behavioural abnormalities, neuroimaging findings of a TCC and white matter abnormalities and evidence of optic atrophy on fundoscopy.

In family 9 (Fig. 1b), we identified a homozygous frameshift deletion in the *CYP2U1* gene (NM_183075.2:c.(782_785delTCTG), NP_898898:p.(Cys262*), at the site of a previously reported pathogenic missense mutation [9]. This variant was heterozygous in both unaffected parents. The clinical features in this family are consistent with the core clinical features of spastic paraplegia, including an early age at onset (<8 years), developmental delay and spastic gait.

We did not find any likely pathogenic variants in known HSP genes in family 1 (Fig. 1c). We did, however, identify a homozygous in-frame deletion in the *PEX16* gene (NM_004813.2:c.(995_997delTCT), NP_004804.1:p.(Phe332del)). Mutations in *PEX16* can cause peroxisomal biogenesis disorder 8A (Zellweger, OMIM 614876) and 8B (OMIM 614877), for which this variant has been reported as likely pathogenic (ClinVar 209181). The same variant also in a homozygous state was recently identified in a patient with progressive ataxia and a mild elevation of very long-chain fatty acids (VLCFAs) [10]. Neuroimaging findings in the proband from family 1 include white matter abnormalities and cervical cord atrophy, consistent with a peroxisomal disorder (see inset, Fig. 1c).

For family 7 (Fig. 1d), no convincing candidate variants were identified in known HSP genes to explain the severe phenotype which included spastic limbs, limb dystonia and developmental delay. We subsequently identified compound heterozygous variants in the *GLB1* gene, known to cause GM1 gangliosidosis (OMIM 230500) in the affected siblings. This included a paternally inherited, novel canonical splice site variant (NM_000404.2:c.(553-2A>G)) and a maternally inherited previously reported pathogenic variant (NM_000404.2:c.(1325G>A); NP_000395:p.(Arg442Gln)) [11]. Enzymology for GM1 gangliosidosis was subsequently performed on peripheral blood leukocytes as described [12], confirming reduced β-galactosidase enzyme activity of 1.6 (normal range 32.5–206.5 nmol/h/mg protein). Clinical evaluation did not detect any evidence of skeletal involvement, cardiac involvement or hepatosplenomegaly.

**Fig. 2:**
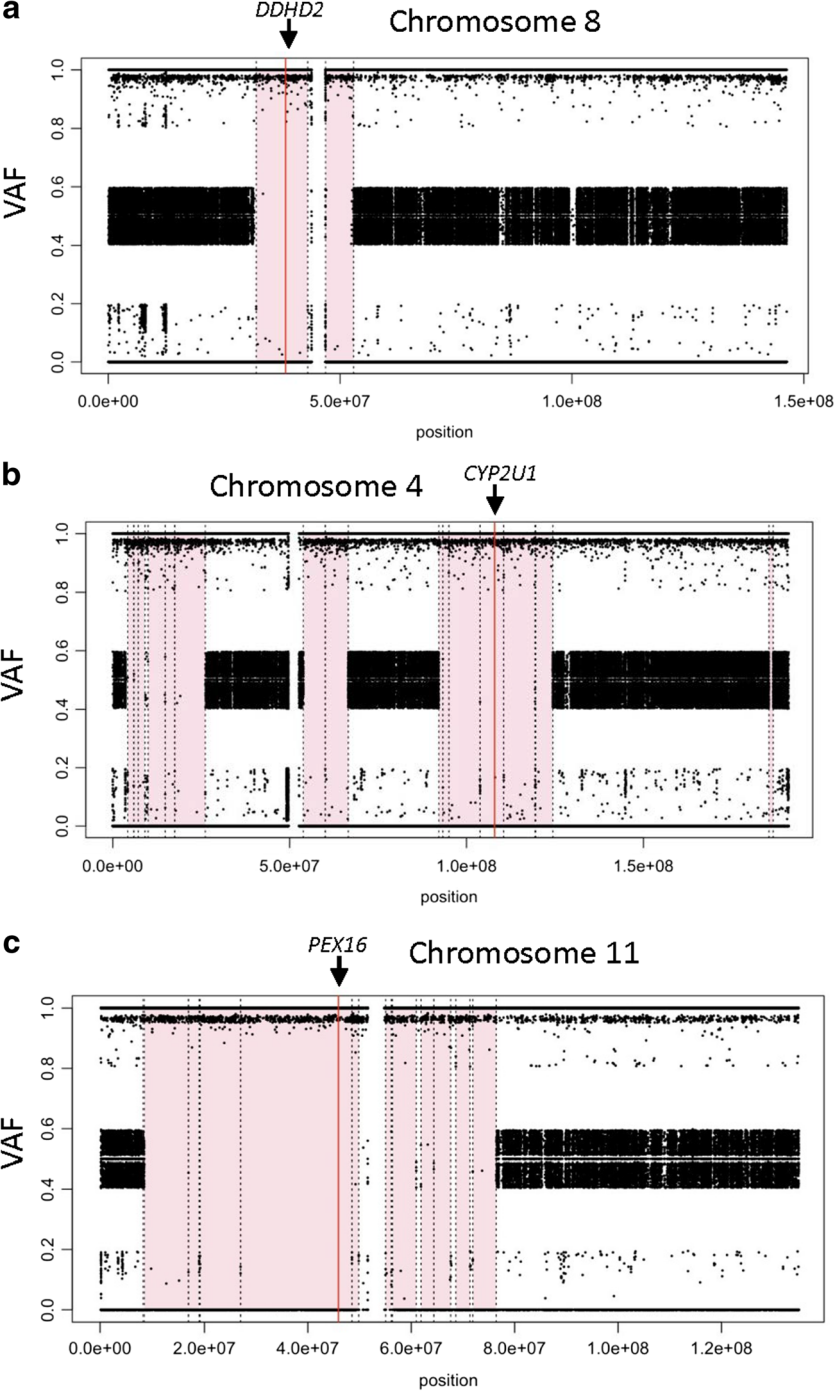
Homozygosity mapping using PLINK confirmed that putative homozygous variants were located within regions of homozygosity (*shaded pink*) for affected members from family 12, family 9 and family 1 (panels **a**–**c**, respectively). *VAF* variant allele frequency

We developed ROHmer to perform homozygosity mapping from WGS data, which confirmed that all homozygous mutations were located within regions of homozygosity (Fig. 2, Supplementary Fig. 3).

Five families remained undiagnosed, i.e. families 3, 5, 6, 8 and 11 (Supplementary Fig. 4), four of which we only sequenced the proband and two were consanguineous. We searched an expanded set of 589 predicted HSP genes (HSPome [5]), and used dedicated analysis to identify CNV, SV (Supplementary Fig. 5) and predicted splice variants that affect HSP genes. No additional promising candidates were identified.

## Discussion

We identified a genetic diagnosis in 4/9 (~44 %) families with HSP. A genetic cause was detected in those cases in which multiple family members were sequenced (4/4), and those that were consanguineous (4/6). In two families, we identified novel homozygous variants in established SPG genes (*DDHD2* and *CYP2U1*), consistent with the previously described phenotype. In a further two families, a genetic aetiology was identified in non-SPG genes, leading to novel genotype-phenotype associations related to neurometabolic disorders. In family 1, we identified an in-frame deletion in *PEX16*. This mutation has been reported in a patient with spastic ataxia and white matter abnormalities on MRI [10]. The proband from our study was considered to have a HSP rather than a form of ‘spastic ataxia’ given the predominance of lower limb weakness and spasticity. This finding provides additional evidence that perixosomes play a role in the pathogenesis of HSP [13].

We identified compound heterozygous variants in the *GLB1* gene in family 7. The phenotype overlapped with GM1 gangliosidosis type II (late infantile). This suggests that GM1 gangliosidosis may be a phenocopy for a severe, early-onset, complicated form of HSP. In this case, hypothesis-free genetic testing prompted the clinician to reassess the patient leading to a change of diagnosis, reminiscent of the ‘reverse phenotyping’ approach reported previously [14]. The identification of unexpected neurometabolic disorders in this study highlights that a broader metabolic work-up may be valid in patients presenting with early-onset HSP.

WGS allowed us to perform a number of additional investigations that would not have been possible with targeted approaches, including dedicated analysis for CNVs, SVs and splice site prediction deep within introns. This multimodal approach should be investigated further using larger sample sizes.

In 5/9 families, a genetic diagnosis was not identified. Without additional family members, prioritising variants outside of the HSP genes is challenging. It is possible that pathogenic variants in HSP genes were missed due to suboptimal coverage, or that variants fell in regions that are difficult to interpret (introns, untranslated or regulatory regions). Furthermore, triplet-repeat disorders can overlap with the HSP phenotype, and may not be readily identifiable from WGS data. Moreover, in sporadic cases, acquired causes should also be considered.

This study is consistent with early-onset HSP phenotypes being secondary to pathogenic variants in both SPG and in non-SPG genes, with consideration of inherited peroxisomal or lysosomal disorders being important phenocopies. If a targeted sequencing approach assessing only known SPG genes had been taken, the diagnostic rate would have been just 20 %. Rather than undertaking a series of cascading genetic tests as part of a diagnostic odyssey, WES or WGS could be used as the initial genetic test for patients with early-onset HSP. It may be particularly effective when used in the context of a family study or in populations where the mutation spectrum is unknown or difficult to anticipate.

## Electronic supplementary material

ESM 1 (DOCX 3524 kb)

ESM 2 (DOCX 97 kb)
