# Supplementary material for: Defining the genetic basis of early onset hereditary spastic paraplegia using whole genome sequencing
Source: Neurogenetics. 2016 Sep 28;17(4):265–70. doi: 10.1007/s10048-016-0495-z (PMC5061846; doi:10.1007/s10048-016-0495-z)
Supplement: Supplementary file 1 — (DOCX 3524 kb) [file 10048_2016_495_MOESM1_ESM.docx]

Defining the Genetic Basis of Early Onset Hereditary Spastic Paraplegia Using Whole Genome Sequencing

Kishore R Kumar, MBBS, PhD, FRACP;^1,2^* G.M. Wali, MD, DM;*^3^ Mahesh Kamate, MD, DM;*^4^ Gautam Wali, PhD;^1^ André E Minoche, PhD;^2^ Clare Puttick, BSc (Hons);^2^ Mark Pinese, PhD;^2^ Velimir Gayevskiy, PhD;^2^ Marcel E Dinger, PhD;^2^ Tony Roscioli, MBBS, PhD, FRACP;^1,5,6^* Carolyn M. Sue, MBBS, FRACP, PhD;^1^* Mark J Cowley, PhD^2,5^*

**SUPPLEMENTARY FIGURES**


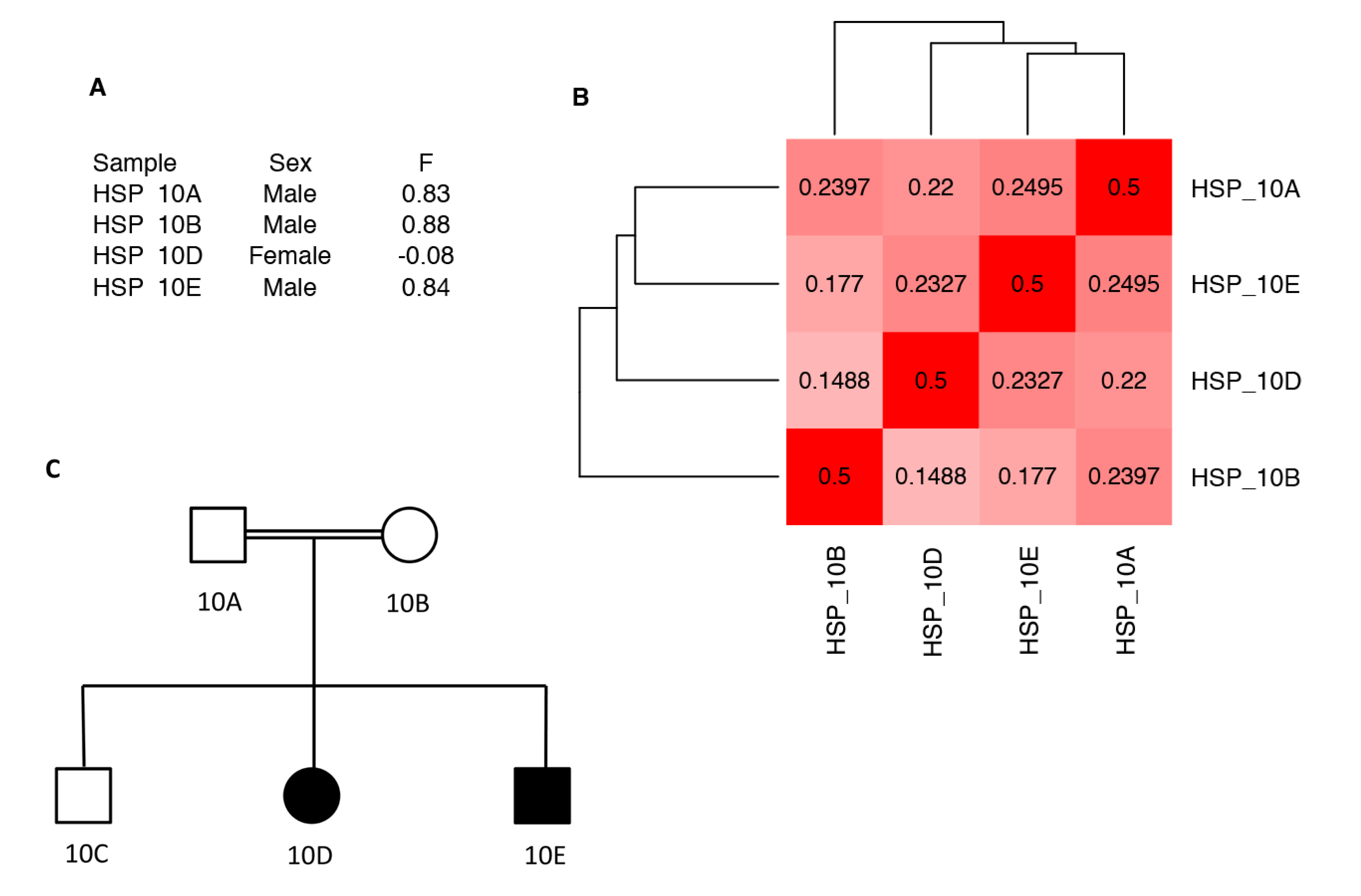


Supplementary Figure 1: Gender and Relatedness checks failed for Family 10. We predict gender (A) and relatedness (B), compared to the expected pedigree (C). To determine the gender of each sample (A), we use PLINK, which compares the zygosity of variants on the X-chromosome. Default settings were used, where F score > 0.8 are male, and < 0.2 are female. To assess relatedness between samples (B), we calculated the kinship coefficient of relatedness between all pairwise combinations of individuals using KING. Scores of 0.5 represent self-self, or monozygotic-twin comparisons, whereas 0.25 and 0.125 represent first, and second-degree relatives, respectively. Gender and relatedness of all other families in this cohort passed check (data not shown).


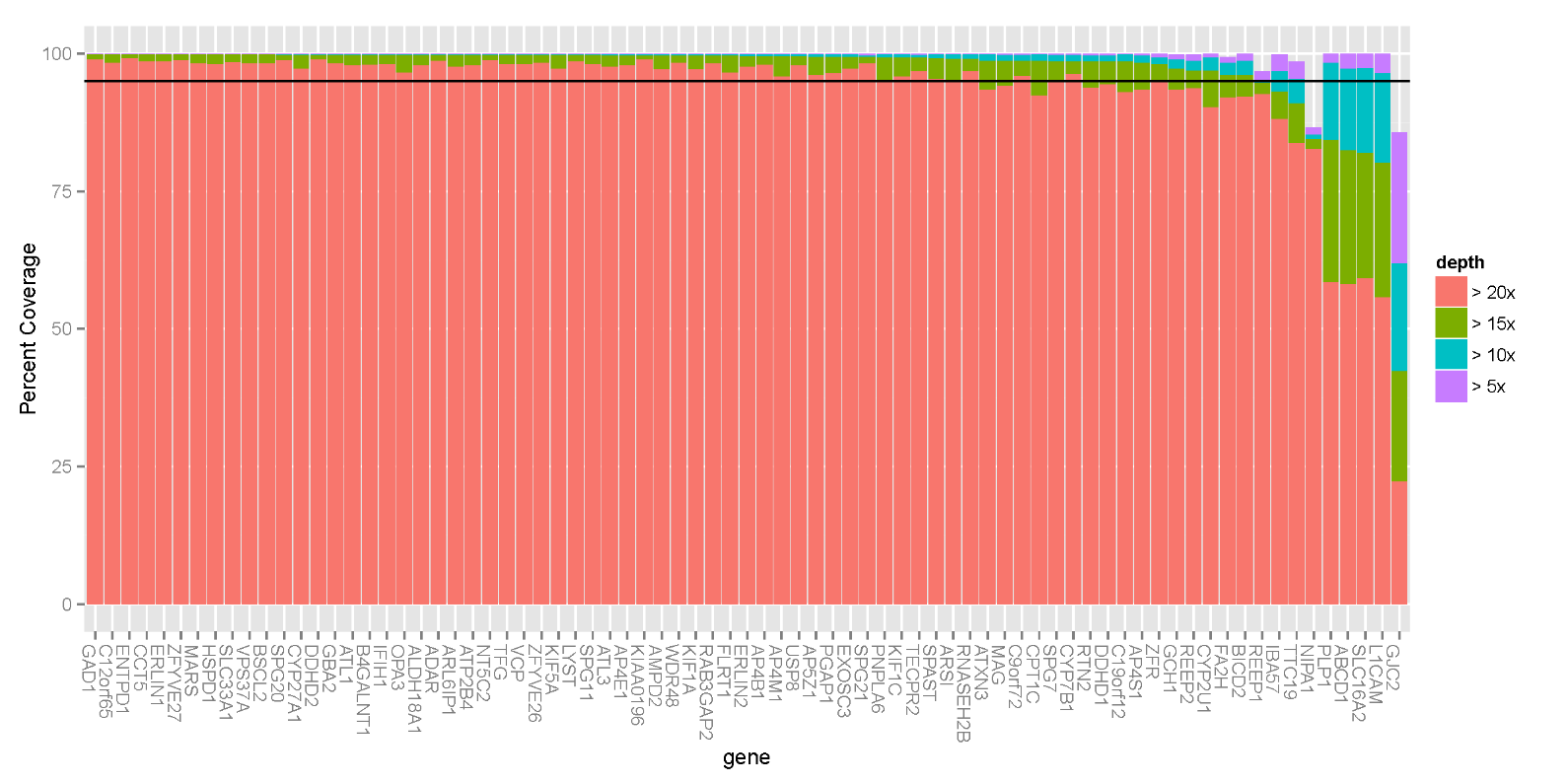


Supplementary Figure 2. Depth of sequencing coverage for each of the 77 nuclear genes associated with HSP. The percentage of coding bases within each gene, that has sequence coverage greater than the depths indicated in the legend are reported. Black horizontal line represents 95%.

Supplementary Figure 3. Runs of homozygosity identified using PLINK in diagnosed HSP families. The red line indicates that the putative causative gene lies within a region of homozygosity in affected family members. A. Family 12, B. Family 9, C. Family 1.


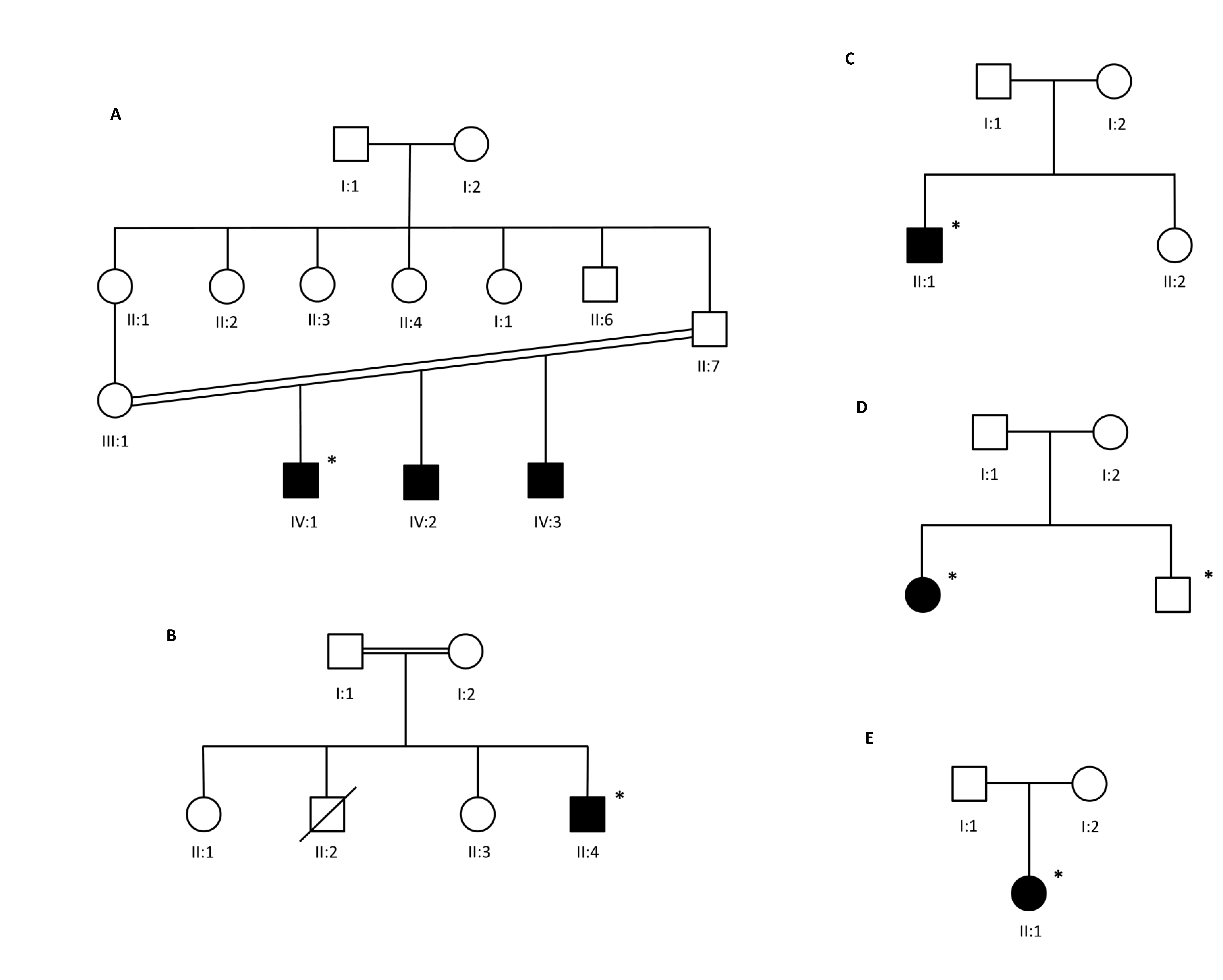


Supplementary Figure 4. Pedigrees of undiagnosed HSP families. A. Family 3, B. Family 5, C. Family 6, D. Family 8, E. Family 11. Asterisk, patient underwent WGS.


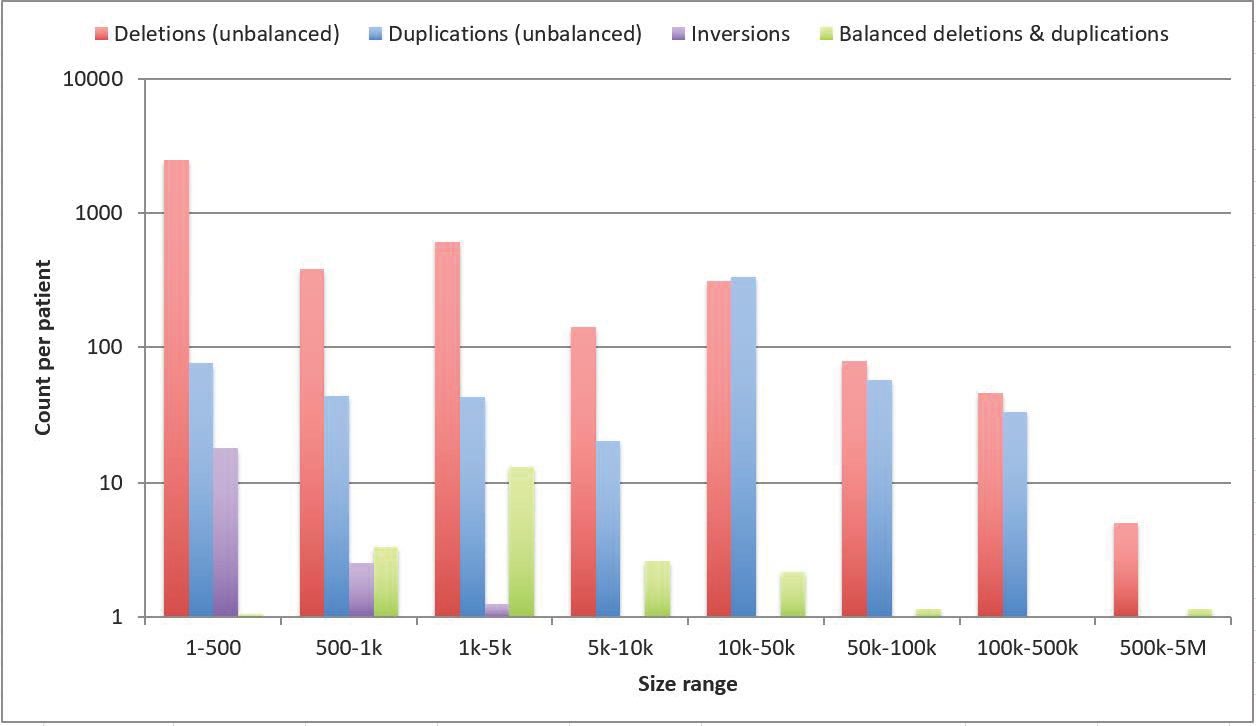


Supplementary Figure 5. The average number of structural variants and copy number variants across the undiagnosed individuals is reported across different size ranges.
