# Supplementary material for: Defining the genetic basis of early onset hereditary spastic paraplegia using whole genome sequencing
Source: Neurogenetics. 2016 Sep 28;17(4):265–70. doi: 10.1007/s10048-016-0495-z (PMC5061846; doi:10.1007/s10048-016-0495-z)
Supplement: Supplementary file 2 — (DOCX 97 kb) [file 10048_2016_495_MOESM2_ESM.docx]

Defining the Genetic Basis of Early Onset Hereditary Spastic Paraplegia Using Whole Genome Sequencing

Kishore R Kumar, MBBS, PhD, FRACP;^1,2^* G.M. Wali, MD, DM;*^3^ Mahesh Kamate, MD, DM;*^4^ Gautam Wali, PhD;^1^ André E Minoche, PhD;^2^ Clare Puttick, BSc (Hons);^2^ Mark Pinese, PhD;^2^ Velimir Gayevskiy, PhD;^2^ Marcel E Dinger, PhD;^2^ Tony Roscioli, MBBS, PhD, FRACP;^1,5,6^* Carolyn M. Sue, MBBS, FRACP, PhD;^1^* Mark J Cowley, PhD^2,5^*

**SUPPLEMENTARY FILE**

**EXTENDED METHODS**

**WGS and Bioinformatic Filtering**

Sequencing libraries were prepared using robotic instrumentation and sequenced on the Illumina HiSeq X sequencers at the Kinghorn Centre for Clinical Genomics (KCCG), with 2x150 b.p. reads, yielding at least 110 Gb of raw sequence data per lane. Data were analysed following the GATK best practices pipeline, as previously described in detail [[1](#_ENREF_1)]. Briefly, reads were aligned to the b37d5 reference genome using BWA MEM, and novosort. Reads were realigned around known indels, and base scores recalibrated using GATK. SNVs and indels were identified using GATK HaplotypeCaller. Samples were joint-called in two batches according to their processing date. Variant filters were assessed using VQSR, based on standard covariates. Variants were annotated with VEP and then converted into a Gemini database. Read depth was assessed using GATK DepthOfCoverage, over RefSeq genes. All samples underwent gender and relatedness checks, using PLINK v1.90b1g REF, and KING v1.4 [[2](#_ENREF_2)], respectively. Families failing these checks were not considered for further investigation.

Filtering of variants was performed using Seave, an in-house variant filtering platform. After filtering variants that PASS VQSR filters, they were prioritised based on rarity (<1%) in the 1000 genomes [[3](#_ENREF_3)], the Exome Sequencing Project (ESP) 6500 exomes, and the Exome Aggregation Consortium (ExAC) database. Furthermore, candidate variants were prioritised according to whether they were present and segregated with disease in unrelated pedigrees from the Indian HSP sample. Variants were filtered according to Gemini Database Schema criteria for impact as either high (e.g. nonsense, frameshift, splice acceptor, splice donor) or medium (e.g. missense, inframe codon insertions or deletions) [[4](#_ENREF_4)]. Missense variants were further prioritised according to predicted pathogenicity on at least 1 of 2 software prediction programs [SIFT (<http://sift.jcvi.org/>) and Polyphen2 (<http://genetics.bwh.harvard.edu/pph2/>), accessed Jan 2016]. Initial priority was given to variants in known HSP genes (Supplementary Table 1, includes comparison with commercially available HSP panels) and genes known to cause neurological phenotypes. We expanded the search space to the HSPome, a database of 589 genes putatively associated with HSP [[5](#_ENREF_5)]. For the family studies, variants were also filtered according to a homozygous, compound heterozygous, *de novo*, and X-linked recessive model of inheritance, as appropriate according to family history. We investigated genetic variants of the mitochondrial gene *MT-ATP6* using HaplotypeCaller, which is able to detect variants of >20% heteroplasmy (data not shown).

Putative pathogenic variants were confirmed by Sanger sequencing (see previous publications for primer sequences [[6-9](#_ENREF_6)]) on an ABI3100 (BigDye® Xterminator Kit, Applied Biosystems). Novel variants were classified according to American College of Medical Genetics and Genomics (ACMG) 2015 guidelines [[10](#_ENREF_10)].

**Homozygosity Mapping**

We identified contiguous homozygous regions directly from WGS data, for each consanguineous family separately. The broad coverage of WGS yielded approximately six million SNVs per family, which permitted the use of stringent variant quality filters while still maintaining excellent genomic resolution. Variants were filtered using the package VariantAnnotation [[11](#_ENREF_11)] (v1.12.9) in R (v3.1.3), by retaining only SNVs with dbSNP ID’s, depth > 20 and < 120 (i.e. 4x the average autosomal read depth), and genotype quality > 30, that were located outside of repetitive regions defined by RepeatMasker (Smit, AFA, Hubley, R & Green, P. *RepeatMasker Open-4.0*. 2013-2015 http://www.repeatmasker.org, accessed Jan 2016). Any variants with skewed allelic depth were removed by requiring variant allele frequencies to fall in the ranges of 0-0.2, 0.4-0.6, or 0.8-1. From these filtered variants, homozygosity mapping was performed using the runs of homozygosity tool within PLINK [[12](#_ENREF_12)] (v1.07). We used the following default settings for PLINK: a sliding window of 50 SNPs across the genome was used to detect long contiguous runs of homozygous genotypes, allowing up-to 1 heterozygous SNP per window; the minimum proportion of non-homozygous windows overlapping a SNP was 0.05 for the SNP to be considered homozygous. Potential pathogenic variants were intersected with regions of homozygosity in each family for further review.

**Detection of Structural Variants/Copy Number Variants**

Read alignment bam files were used as input to call structural variation with LUMPY [[13](#_ENREF_13)] (v0.2.11, settings -mw 4, -tt 0, -x, -SR back_distance:10, weight:1, min_mapping_threshold:20 -PE discordant_z:4, back_distance:10, weight:1, min_mapping_threshold:20) and CNVs with CNVNator (v0.3[[14](#_ENREF_14)]) multithreaded (v.0.0.1[[15](#_ENREF_15)] default settings). Uncharacterized breakends (BNDs) were required to have at least 6 lines of evidence from discordant pairs or split reads. CNVNator calls were required to have a minimum length of 10kb. Deletions showing no copy number change but compensating tandem-duplications were classified as balanced deletion-duplication events. To reduce the number of non-genuine balanced events, due to ambiguously mapping reads, balanced events were required to have an average mapping quality of 60 at breakpoints. LUMPY and CNVnator calls of the same type (unbalanced deletions and duplications) were merged if they showed a reciprocal overlap length of at least 90%. Overlaps between the merged CNV calls and known HSP genes (78 genes) were assessed. Candidate variant calls and the corresponding read-alignments were inspected using the Integrative Genomics Viewer [[16](#_ENREF_16)]. Variant calls having no clear breakpoints were discarded.

**Detection of Aberrant Splicing**

We used the offline version of SPANR [[17](#_ENREF_17)], known as SPIDEX, to assess for single nucleotide variations (SNVs) cause splicing misregulation and that may lead to disease. SPANR uses machine learning over hundreds of features to learn the typical characteristics of spliced exons, and can predict the impact of any coding or intronic SNVs within 150 b.p. or 300 b.p. of the splice junction, respectively. We restricted the SPIDEX predictions to known HSP genes, and converted them to a VCF file. We annotated variants from the cohort with GATK VariantAnnotator, and followed up any top ranking variants that were rare and matched the inheritance pattern for each family using the SPANR web-tool.

**TABLES**

Supplementary Table 1. We curated a set of 78 known HSP genes, some of which are offered by commercial testing panels as indicated (accessed online Feb 2016). The asterisk (*) indicates that deletion/duplication testing is also offered by commercial laboratories as a separate test. We refer to 77 HSP genes throughout the main text, which excludes MT-ATP6, which is in the mitochondria. UA, unassigned; AD, autosomal dominant; AR, autosomal recessive; XLD, x linked dominant; XLR, x linked recessive; mt, mitochondrial.

| HSP Locus | Gene Name | Reported mode of inheritance | Centogene: Spastic Paraplegia panel AD or AR | Liverpool RGC: Hereditary Spastic Paraplegia 20 Gene Panel | GeneDx: Comprehensive Hereditary Spastic Paraplegia Panel |
| --- | --- | --- | --- | --- | --- |
| SPG1 | *L1CAM* | XLR |  | ✓ | ✓ |
| SPG2 | *PLP1* | XLR | ✓* | ✓ | ✓ |
| SPG3A | *ATL1* | AD | ✓* | ✓ | ✓ |
| SPG4 | *SPAST* | AD | ✓* | ✓ | ✓ |
| SPG5A | *CYP7B1* | AR | ✓ | ✓ | ✓ |
| SPG6 | *NIPA1* | AD | ✓* | ✓ | ✓ |
| SPG7 | *SPG7* | AR | ✓* | ✓ | ✓ |
| SPG8 | *KIAA0196* | AD | ✓ | ✓ | ✓ |
| SPG9A | *ALDH18A1* | AD |  |  |  |
| SPG10 | *KIF5A* | AD | ✓ | ✓ | ✓ |
| SPG11 | *SPG11* | AR | ✓* | ✓ | ✓ |
| SPG12 | *RTN2* | AD | ✓ |  | ✓ |
| SPG13 | *HSPD1* | AD | ✓ | ✓ |  |
| SPG15 | *ZFYVE26* | AR | ✓ | ✓ | ✓ |
| SPG17 | *BSCL2* | AD | ✓ | ✓ | ✓ |
| SPG18 | *ERLIN2* | AR | ✓ |  |  |
| SPG20 | *SPG20* | AR | ✓ | ✓ | ✓ |
| SPG21 | *SPG21* | AR | ✓ | ✓ | ✓ |
| SPG22 | *SLC16A2* | XLD |  |  | ✓ |
| SPG26 | *B4GALNT1* | AR | ✓ |  | ✓ |
| SPG28 | *DDHD1* | AR | ✓ |  | ✓ |
| SPG30 | *KIF1A* | AD and AR | ✓ |  | ✓ |
| SPG31 | *REEP1* | AD | ✓* | ✓ | ✓ |
| SPG33 | *ZFYVE27* | AD | ✓ |  |  |
| SPG35 | *FA2H* | AR | ✓ | ✓ | ✓ |
| SPG39 | *PNPLA6* | AR | ✓ |  | ✓ |
| SPG42 | *SLC33A1* | AD | ✓ | ✓ |  |
| SPG43 | *C19orf12* | AR | ✓ |  |  |
| SPG44 | *GJC2* | AR | ✓ | ✓ | ✓ |
| SPG46 | *GBA2* | AR | ✓ |  | ✓ |
| SPG47 | *AP4B1* | AR | ✓ |  | ✓ |
| SPG48 | *AP5Z1* | AR | ✓ |  |  |
| SPG49 | *TECPR2* | AR | ✓ |  | ✓ |
| SPG50 | *AP4M1* | AR | ✓ |  | ✓ |
| SPG51 | *AP4E1* | AR | ✓ |  | ✓ |
| SPG52 | *AP4S1* | AR | ✓ |  | ✓ |
| SPG53 | *VPS37A* | AR | ✓ |  | ✓ |
| SPG54 | *DDHD2* | AR | ✓ |  | ✓ |
| SPG55 | *C12orf65* | AR | ✓ |  | ✓ |
| SPG56 | *CYP2U1* | AR | ✓ |  | ✓ |
| SPG57 | *TFG* | AR | ✓ |  |  |
| SPG58 | *KIF1C* | AR |  |  | ✓ |
| SPG59 | *USP8* | AR | ✓ |  |  |
| SPG60 | *WDR48* | AR | ✓ |  |  |
| SPG61 | *ARL6IP1* | AR | ✓ |  |  |
| SPG62 | *ERLIN1* | AR | ✓ |  | ✓ |
| SPG63 | *AMPD2* | AR | ✓ |  |  |
| SPG64 | *ENTPD1* | AR | ✓ |  |  |
| SPG65 | *NT5C2* | AR | ✓ |  | ✓ |
| SPG66 | *ARSI* | AR | ✓ |  |  |
| SPG67 | *PGAP1* | AR |  |  |  |
| SPG68 | *FLRT1* | AR | ✓ |  |  |
| SPG69 | *RAB3GAP2* | AR |  |  |  |
| SPG70 | *MARS* | AR |  |  |  |
| SPG71 | *ZFR* | AR | ✓ |  |  |
| SPG72 | *REEP2* | AR | ✓ |  |  |
| SPG73 | [*CPT1C*](http://www.genenames.org/cgi-bin/gene_symbol_report?match=CPT1C) | AD |  |  |  |
| SPG74 | *IBA57* | AR |  |  |  |
| SPG75 | *MAG* | AR |  |  |  |
| UA | *GAD1* | AR |  |  |  |
| UA | *CCT5* | AR | ✓ |  |  |
| UA | *OPA3* | AR |  |  |  |
| UA | *BICD2* | AD |  |  |  |
| UA | *LYST* | AR |  |  |  |
| UA | *MT-ATP6* | Mt |  |  |  |
| UA | *ABCD1* | XLR |  |  |  |
| UA | *ATXN3* | AD |  |  |  |
| UA | *ATP2B4* | AD |  |  |  |
| UA | *CYP27A1* | AR |  |  |  |
| UA | *TTC19* | AR |  |  |  |
| UA | *ADAR1* | AD |  |  |  |
| UA | *IFIH1* | AD |  |  |  |
| UA | *RNASEH2B* | AR |  |  |  |
| UA | *VCP* | AD |  |  |  |
| UA | *EXOSC3* | AR |  |  |  |
| UA | *C9orf72* | AD |  |  |  |
| UA | *ATL3* | AD |  |  |  |
| UA | *GCH1* | AD |  |  |  |

Supplementary Table 2. Variant counts for affected individuals undergoing WGS. Variant impact (Medium or High) is determined by Gemini, where Medium includes missense variants, and High includes loss of function, frameshift and essential splice variants. SIFT and Polyphen are in silico methods to assess the pathogenicity of missense variants.

|  | Family 11 (II:1) | Family 12 (II:1) | Family 12 (II:2) | Family 5 (II:4) | Family 6 (II:1) | Family 7 (II:2) | Family 7 (II:3) | Family 8 (II:1) | Family 9 (IV3) | Family 1 (V:3) | Family 3 (IV1) |
| --- | --- | --- | --- | --- | --- | --- | --- | --- | --- | --- | --- |
| **All Genes** |  |  |  |  |  |  |  |  |  |  |  |
| Total Passed Variants | 4783027 | 4809625 | 4633668 | 4711735 | 4766023 | 4892224 | 4824132 | 4652934 | 4602549 | 4468559 | 4468559 |
| Medium Impact | 18037 | 18325 | 17427 | 18073 | 18140 | 18480 | 18515 | 18054 | 17501 | 17420 | 17420 |
| Medium Impact Polyphen2 & SIFT Damaging | 417 | 439 | 426 | 419 | 406 | 411 | 454 | 410 | 399 | 529 | 529 |
| High Impact | 1281 | 1275 | 1238 | 1217 | 1272 | 1286 | 1316 | 1273 | 1308 | 1264 | 1264 |
| High Impact Stop Gained or Lost | 216 | 216 | 195 | 195 | 209 | 190 | 197 | 203 | 199 | 207 | 207 |
| High Impact Splice Site | 581 | 565 | 557 | 554 | 585 | 591 | 589 | 579 | 588 | 554 | 554 |
| High Impact Frame Shift | 435 | 454 | 443 | 423 | 436 | 450 | 468 | 437 | 479 | 457 | 457 |
|  |  |  |  |  |  |  |  |  |  |  |  |
| **HSP Genes** |  |  |  |  |  |  |  |  |  |  |  |
| Total Passed Variants | 6911 | 6590 | 5402 | 6370 | 6123 | 6862 | 6247 | 5620 | 5976 | 5837 | 5837 |
| Medium Impact | 51 | 53 | 39 | 51 | 53 | 44 | 45 | 36 | 39 | 48 | 48 |
| Medium Impact Polyphen2 & SIFT Damaging | 1 | 2 | 3 | 2 | 1 | 1 | 1 | 1 | 1 | 1 | 1 |
| High Impact | 3 | 3 | 1 | 3 | 3 | 0 | 1 | 3 | 2 | 3 | 3 |
| High Impact Stop Gained or Lost | 1 | 1 | 0 | 1 | 0 | 0 | 0 | 1 | 0 | 1 | 1 |
| High Impact Splice Site | 0 | 1 | 0 | 0 | 0 | 0 | 0 | 0 | 0 | 0 | 0 |
| High Impact Frame Shift | 2 | 1 | 1 | 2 | 3 | 0 | 1 | 2 | 2 | 2 | 2 |

Supplementary Table 3. Clinical presentation, laboratory investigations, and WGS results for nine Indian families with HSP

| **Family** | **Consanguineous** | **Genogram Position** | **Gender** | **Age of onset** | **Age at examination** | **Clinical Phenotype** | **Investigations** | **Gene** | **Genomic coordinate^¥^** | **Coding change** | **Protein change** | **Homozygous or heterozygous** | **Previous report of pathogenicity, ACMG Criteria for classifying variants** |
| --- | --- | --- | --- | --- | --- | --- | --- | --- | --- | --- | --- | --- | --- |
| 12 | Yes | II1 | M | Infancy | 12 years | Spastic paraplegia, delayed speech, intellectual impairment, behaviour abnormalities, optic atrophy | MRI brain: TCC with paucity of white matter | *DDHD2* | chr8:g.38103819G>T | NM_015214.2:c.1125+1G>T |  | Hom | PVS1 |
|  |  | II2 | F | Infancy | 9 years | Spastic paraplegia, delayed speech, intellectual impairment, behaviour abnormalities | MRI brain: TCC with paucity of white matter |  |  |  |  |  |  |
| 9 | Yes | IV3 | F | Infancy | 4 years | Spastic gait, developmental delay, bladder disturbance | MRI brain: normal  Serum lactate: normal  MS/MS: normal | *CYP2U1* | chr4:g.108866416delTCTG | NM_183075.2:c.782_785delTCTG | [NP_898898](http://www.ncbi.nlm.nih.gov/nuccore/NP_898898);p.Cys262* | Hom | PVS1 |
| 1 | Yes | V3 | M | 11 months | 11 years | Spastic paraplegia, cerebellar ataxia, dysarthria, milestone regression, takes a few steps with support | MRI brain: white matter abnormalities, cerebellar atrophy  MRI spine: cervical cord atrophy  NCS: normal | *PEX16* | chr11:g.45931818delTAGA | NM_004813.2:c.(995_997delTCT) | [NP_004804.1:p.(Phe332](http://www.ncbi.nlm.nih.gov/projects/sviewer/?id=NP_004804.1&search=NP_004804.1:p.Phe332&v=1:100&content=5)del) | Hom | Identified in a patient with progressive ataxia [[18](#_ENREF_18)]. Extensive plasma and fibroblast analysis in the previously reported patient showed subtle peroxisomal biochemical abnormalities.  PS1 |
| 7 | Yes | II1 | F | Infancy | 20 years | Spastic limbs, limb dystonia, developmental delay, marked speech disturbance, unable to mobilise |  | *GLB1* | chr3:g.33059962C>T | NM_000404.2:c.1325G>A | [NP_000395](http://www.ncbi.nlm.nih.gov/nuccore/NP_000395):p.(Arg442Gln) | Het | Reported in an adult GM1 gangliosidosis patient in a compound heterozygous state [[19](#_ENREF_19)].  PS1 |
|  |  | II2 | F | Infancy | 18 years | Spastic limbs, limb dystonia, developmental delay, marked speech disturbance, unable to mobilise |  |  | chr3:g.33099763T>C | NM_000404.2:c.553-2A>G |  | Het | PVS1 |
|  |  | II3 | M | Infancy | 12 years | Spastic gait, limb dystonia, developmental delay, drooling of saliva  No organomegaly or skeletal manifestations | MRI brain: globus pallid hypointensities were evident on repeat evaluation.  β-galactosidase enzyme activity of 1.6 (normal range: 32.5 - 206.5 nmol/hr/mg protein) |  |  |  |  |  |  |
| 3 | Yes | IV1 | M | Infancy | 7 years | Spastic gait, developmental delay, speech disturbance (utters single words only), behavioural abnormalities (irritable, incessant crying), hypersalivation | MRI brain: normal | No candidate variants |  |  |  |  |  |
|  |  | IV2 | M | Infancy | 5 years | Spastic gait, developmental delay, mute, behavioural abnormalities (irritable, incessant crying), lumbar hyperlordosis | MRI brain: normal | DNA not available |  |  |  |  |  |
|  |  | IV3 | M | Infancy | 2 years 6 months | Spastic paraplegia, developmental delay, mild macrocephaly | MRI brain: normal | DNA not available |  |  |  |  |  |
| 5 | Yes | II4 | M | Birth | 4 years | Limb spasticity with deformity, dystonic posturing in arms, born FTND but did not cry soon after birth, seizures at 8 months of age, poor head holding, marked speech disturbance, mild convergent squint, truncal hypotonia | MRI brain: mild cerebellar atrophy  Targeted HSP panel (MEDGENOME) identified a single heterozygous variant (c.1765C>T, p.R589W) in the *DDHD1* gene, a cause of autosomal recessive HSP (SPG28). | No candidate variants identified |  |  |  |  |  |
| 6 | No | II1 | M | Infancy | 6 years | Delayed walking with a spastic gait | MRI brain: normal | No candidate variants identified |  |  |  |  |  |
| 8 | No | II1 | F | 18 months | 9 years | Gradual onset of spastic gait, urinary urgency | MRI brain: normal | No candidate variants identified |  |  |  |  |  |
| 11 | No | II1 | F | Infancy | 5 years | Spastic paraplegia, microcephaly, seizures, tongue clicking, drooling of saliva | MRI brain: cerebellar atrophy  MS/MS: normal  Serum lactate and ammonia: normal  Tripeptidyl peptidase 1: normal (testing for neuronal ceroid lipofuscinoses type 2) | *SPAST* | chr2:g.32329003G>Achr2:g.32329003G>A | NM_014946.3:c.641A>G | NP_055761.2: p.(Asp214Gly) | Het | Variant identified in heterozygous state in asymptomatic parents.  BS2 |

ACMG, American College of Medical Genetics and Genomics; HGVSc, Human Genome Variation Society nomenclature; M, male; F, female; Het, heterozygous; Hom, homozygous; FTND, full term normal delivery; MRI, magnetic resonance imaging; MS/MS, tandem mass spectroscopy; NCS, nerve conduction studies; TCC, Thin corpus callosum. The term ‘infancy’ is used for onset prior to 12 months of age. ^¥^Reference genome was the 1000 Genomes Phase 2 reference genome (hs37d5), which comprises the GRCh37 reference genome, including decoy sequences and the human herpesvirus 4 type 1.

Supplementary Table 4. Initial analysis in all families for rare variants in the known HSP genes.

| Family | Inheritance of variant in affected probands | Gene | Variant | Variant type | Codon Change | Amino acid change | rs id | SIFT prediction | Polyphen prediction | ESP6500 | 1000 Genomes | ExAC | OMIM Disorders, mode of Inheritance |
| --- | --- | --- | --- | --- | --- | --- | --- | --- | --- | --- | --- | --- | --- |
| 12 | **Hom** | ***DDHD2*** | **chr8:g.38103819G>T** | **Splice donor variant** | **None** | **None** |  | **None** | **None** | **0** | **0** | **0** | **SPG54, autosomal recessive** |
|  | Compound Het | *LYST* | chr1:g.235926124C>T | Missense | cGg/cAg | R/Q | rs376311093 | Tolerated - low confidence | Benign | 0.000154 | 0.000199681 | 0.000138 | Chediak-Higashi syndrome, autosomal recesive |
|  |  |  | chr1:g.235972679A>G | Missense | aTg/aCg | M/T | None | Tolerated | Benign | None | None | None |  |
| 9 | **Hom** | ***CYP2U1*** | **chr4:g.108866415ATCTG>A** | **Frameshift deletion** | **aTCTGt/at** | **IC/X** | **None** | **None** | **None** | **None** | **None** | **None** | **SPG56, autosomal recessive** |
|  | Hom | *KIF1C* | chr17:g.4927256G>A | Missense | cGg/cAg | R/Q | rs201887996 | Tolerated - low confidence | Benign | None | 0.001398 | 0.000505 | Spastic ataxia 2, autosomal recessive |
| 3 | Het | *AP4E1* | chr15:g.51260533C>T | Missense | cCt/cTt | P/L | None | deleterious | Probably damaging | None | 0.000199681 | 0.000106 | SPG51, autosomal recessive |
| 5 | Het | *ZFYVE26* | chr14:g.68274253C>T | Missense | Gag/Aag | E/K | rs200340910 | tolerated | Probably damaging | 0.000154 | 0.003195 | 0.002301 | SP15, autosomal recessive |
|  | Het | *B4GALNT1* | chr12:g.58022914T>C | Missense | gAg/gGg | E/G | rs149670131 | Tolerated | Benign | 0.001076 | 0.0002 | 0.000838 | SPG26, autosomal recessive |
|  | Het | *DDHD1* | chr14:g.53529662G>A | Missense | Cgg/Tgg | R/W | None | Deleterious | Probably damaging | None | None | 0.00004066 | SPG28, autosomal recessive |
|  | Het | *REEP1* | chr2:g.86459761G>A | Missense | gCg/gTg | A/V | None | Tolerated - low confidence | unknown | None | 0.002196 | 0.0008458 | SPG31, autosomal dominant;?Neuronopathy, distal hereditary motor, type VB |
| 6 | Het | *REEP2* | chr5:g.137781274C>T | Missense | gCg/gTg | A/V | rs145269051 | Tolerated | Benign | 0.000691988313086 | None | 0.000187 | SPG72, autosomal recessive, autosomal dominant |
| 8 | Het | *FLRT1* | chr11:g.63885289T>C | Missense | gTa/gCa | V/A | None | Tolerated | Benign | None | None | None | None |
|  | Het | *LYST* | chr1:g.235914551G>A | Missense | gCt/gTt | A/V | None | Tolerated - low confidence | Benign | None | None | 0.00004879 | Chediak-Higashi syndrome, autosomal recessive |
|  | Het | *ZFYVE26* | chr14:g.68272021C>A | Missense | gGc/gTc | G/V | rs35512910 | Tolerated | Benign | 0.002691065662 | 0.00319489 | 0.003692 | SPG15, autosomal recessive |
| 11 | Het | *USP8* | chr15:g.50769530C>T | Missense | aCg/aTg | T/M | None | Tolerated | Benign | None | 0.00259585 | 0.001626 | None |
|  | **Het** | ***SPAST*** | **chr2:g.32323919A>G** | **Missense** | **gAc/gGc** | **D/G** | **None** | **Deleterious** | **Benign** | **None** | **None** | **None** | **SPG4, autosomal dominant** |

Variants of interest are listed in bold. Reference genome was the 1000 Genomes Phase 2 reference genome, which comprises the GRCh37 reference genome, including decoy sequences and the human herpesvirus 4 type 1. Allele frequencies for the ESP6500, 1000 genomes and ExAC databases are listed.

**REFERENCES**

1. Mallawaarachchi AC, Hort Y, Cowley MJ, McCabe MJ, Minoche A, Dinger ME, Shine J, Furlong TJ (2016) Whole-genome sequencing overcomes pseudogene homology to diagnose autosomal dominant polycystic kidney disease. European journal of human genetics : EJHG. doi:10.1038/ejhg.2016.48

2. Manichaikul A, Mychaleckyj JC, Rich SS, Daly K, Sale M, Chen WM (2010) Robust relationship inference in genome-wide association studies. Bioinformatics 26 (22):2867-2873. doi:10.1093/bioinformatics/btq559

3. Genomes Project C, Abecasis GR, Auton A, Brooks LD, DePristo MA, Durbin RM, Handsaker RE, Kang HM, Marth GT, McVean GA (2012) An integrated map of genetic variation from 1,092 human genomes. Nature 491 (7422):56-65. doi:10.1038/nature11632

4. Paila U, Chapman BA, Kirchner R, Quinlan AR (2013) GEMINI: integrative exploration of genetic variation and genome annotations. PLoS computational biology 9 (7):e1003153. doi:10.1371/journal.pcbi.1003153

5. Novarino G, Fenstermaker AG, Zaki MS, Hofree M, Silhavy JL, Heiberg AD, Abdellateef M, Rosti B, Scott E, Mansour L, Masri A, Kayserili H, Al-Aama JY, Abdel-Salam GM, Karminejad A, Kara M, Kara B, Bozorgmehri B, Ben-Omran T, Mojahedi F, Mahmoud IG, Bouslam N, Bouhouche A, Benomar A, Hanein S, Raymond L, Forlani S, Mascaro M, Selim L, Shehata N, Al-Allawi N, Bindu PS, Azam M, Gunel M, Caglayan A, Bilguvar K, Tolun A, Issa MY, Schroth J, Spencer EG, Rosti RO, Akizu N, Vaux KK, Johansen A, Koh AA, Megahed H, Durr A, Brice A, Stevanin G, Gabriel SB, Ideker T, Gleeson JG (2014) Exome sequencing links corticospinal motor neuron disease to common neurodegenerative disorders. Science 343 (6170):506-511. doi:10.1126/science.1247363

6. Tesson C, Nawara M, Salih MA, Rossignol R, Zaki MS, Al Balwi M, Schule R, Mignot C, Obre E, Bouhouche A, Santorelli FM, Durand CM, Oteyza AC, El-Hachimi KH, Al Drees A, Bouslam N, Lamari F, Elmalik SA, Kabiraj MM, Seidahmed MZ, Esteves T, Gaussen M, Monin ML, Gyapay G, Lechner D, Gonzalez M, Depienne C, Mochel F, Lavie J, Schols L, Lacombe D, Yahyaoui M, Al Abdulkareem I, Zuchner S, Yamashita A, Benomar A, Goizet C, Durr A, Gleeson JG, Darios F, Brice A, Stevanin G (2012) Alteration of fatty-acid-metabolizing enzymes affects mitochondrial form and function in hereditary spastic paraplegia. Am J Hum Genet 91 (6):1051-1064. doi:10.1016/j.ajhg.2012.11.001

7. Schuurs-Hoeijmakers JH, Geraghty MT, Kamsteeg EJ, Ben-Salem S, de Bot ST, Nijhof B, van de V, II, van der Graaf M, Nobau AC, Otte-Holler I, Vermeer S, Smith AC, Humphreys P, Schwartzentruber J, Consortium FC, Ali BR, Al-Yahyaee SA, Tariq S, Pramathan T, Bayoumi R, Kremer HP, van de Warrenburg BP, van den Akker WM, Gilissen C, Veltman JA, Janssen IM, Vulto-van Silfhout AT, van der Velde-Visser S, Lefeber DJ, Diekstra A, Erasmus CE, Willemsen MA, Vissers LE, Lammens M, van Bokhoven H, Brunner HG, Wevers RA, Schenck A, Al-Gazali L, de Vries BB, de Brouwer AP (2012) Mutations in DDHD2, encoding an intracellular phospholipase A(1), cause a recessive form of complex hereditary spastic paraplegia. Am J Hum Genet 91 (6):1073-1081. doi:10.1016/j.ajhg.2012.10.017

8. Bidchol AM, Dalal A, Trivedi R, Shukla A, Nampoothiri S, Sankar VH, Danda S, Gupta N, Kabra M, Hebbar SA, Bhat RY, Matta D, Ekbote AV, Puri RD, Phadke SR, Gowrishankar K, Aggarwal S, Ranganath P, Sharda S, Kamate M, Datar CA, Bhat K, Kamath N, Shah H, Krishna S, Gopinath PM, Verma IC, Nagarajaram HA, Satyamoorthy K, Girisha KM (2015) Recurrent and novel GLB1 mutations in India. Gene 567 (2):173-181. doi:10.1016/j.gene.2015.04.078

9. Ebberink MS, Csanyi B, Chong WK, Denis S, Sharp P, Mooijer PA, Dekker CJ, Spooner C, Ngu LH, De Sousa C, Wanders RJ, Fietz MJ, Clayton PT, Waterham HR, Ferdinandusse S (2010) Identification of an unusual variant peroxisome biogenesis disorder caused by mutations in the PEX16 gene. J Med Genet 47 (9):608-615. doi:10.1136/jmg.2009.074302

10. Richards S, Aziz N, Bale S, Bick D, Das S, Gastier-Foster J, Grody WW, Hegde M, Lyon E, Spector E, Voelkerding K, Rehm HL, Committee ALQA (2015) Standards and guidelines for the interpretation of sequence variants: a joint consensus recommendation of the American College of Medical Genetics and Genomics and the Association for Molecular Pathology. Genet Med 17 (5):405-424. doi:10.1038/gim.2015.30

11. Obenchain V, Lawrence M, Carey V, Gogarten S, Shannon P, Morgan M (2014) VariantAnnotation: a Bioconductor package for exploration and annotation of genetic variants. Bioinformatics 30 (14):2076-2078. doi:10.1093/bioinformatics/btu168

12. Purcell S, Neale B, Todd-Brown K, Thomas L, Ferreira MA, Bender D, Maller J, Sklar P, de Bakker PI, Daly MJ, Sham PC (2007) PLINK: a tool set for whole-genome association and population-based linkage analyses. American journal of human genetics 81 (3):559-575. doi:10.1086/519795

13. Layer RM, Chiang C, Quinlan AR, Hall IM (2014) LUMPY: a probabilistic framework for structural variant discovery. Genome biology 15 (6):R84. doi:10.1186/gb-2014-15-6-r84

14. Abyzov A, Urban AE, Snyder M, Gerstein M (2011) CNVnator: an approach to discover, genotype, and characterize typical and atypical CNVs from family and population genome sequencing. Genome research 21 (6):974-984. doi:10.1101/gr.114876.110

15. Chiang C, Layer RM, Faust GG, Lindberg MR, Rose DB, Garrison EP, Marth GT, Quinlan AR, Hall IM (2015) SpeedSeq: ultra-fast personal genome analysis and interpretation. Nature methods 12 (10):966-968. doi:10.1038/nmeth.3505

16. Robinson JT, Thorvaldsdottir H, Winckler W, Guttman M, Lander ES, Getz G, Mesirov JP (2011) Integrative genomics viewer. Nature biotechnology 29 (1):24-26. doi:10.1038/nbt.1754

17. Xiong HY, Alipanahi B, Lee LJ, Bretschneider H, Merico D, Yuen RK, Hua Y, Gueroussov S, Najafabadi HS, Hughes TR, Morris Q, Barash Y, Krainer AR, Jojic N, Scherer SW, Blencowe BJ, Frey BJ (2015) RNA splicing. The human splicing code reveals new insights into the genetic determinants of disease. Science 347 (6218):1254806. doi:10.1126/science.1254806

18. Bacino C, Chao YH, Seto E, Lotze T, Xia F, Jones RO, Moser A, Wangler MF (2015) A homozygous mutation in identified by whole-exome sequencing ending a diagnostic odyssey. Molecular genetics and metabolism reports 5:15-18. doi:10.1016/j.ymgmr.2015.09.001

19. Caciotti A, Donati MA, d'Azzo A, Salvioli R, Guerrini R, Zammarchi E, Morrone A (2009) The potential action of galactose as a "chemical chaperone": increase of beta galactosidase activity in fibroblasts from an adult GM1-gangliosidosis patient. European journal of paediatric neurology : EJPN : official journal of the European Paediatric Neurology Society 13 (2):160-164. doi:10.1016/j.ejpn.2008.03.004
